# Osteochondral Allograft Transplantation of the Lateral Femoral Trochlea Secondary to Traumatic Injury: A Case Report

**DOI:** 10.7759/cureus.80778

**Published:** 2025-03-18

**Authors:** Logan M Druessel, Fortunato G Padua, Hassan Choudhry, Richard M Miller

**Affiliations:** 1 Department of Orthopedic Surgery, Mercy Health St. Vincent Medical Center, Toledo, USA

**Keywords:** fresh osteochondral allograft, lateral femoral trochlea, osteochondral allograft transplantation, patellar instability, patellofemoral instability, traumatic bone loss

## Abstract

A 23-year-old female presented with traumatic loss of the left lateral femoral trochlea and painful patellofemoral instability. She underwent osteochondral allograft transplantation of her left lateral femoral trochlea. After one week, she was able to stand on her left leg, and at six months, she had good patellofemoral stability, was ambulating, and could complete all daily activities as needed. We present this novel case of patellofemoral instability due to traumatic loss of the lateral femoral trochlea treated with a lateral femoral trochlea osteochondral allograft. Our surgical intervention resolved the patient’s instability, improved her pain, and preserved function in this case.

## Introduction

There is a paucity in the literature regarding the treatment of large traumatic loss of the lateral femoral trochlea. The use of a fresh frozen allograft for the reconstruction of a joint surface with significant loss has been performed in other joints. Specifically, reconstruction of the glenoid with a distal tibia allograft has had good reported outcomes [1-3]. Although fresh osteochondral allograft transplantation (OCA) has been successfully performed in patients with femoral trochlear dysplasia or patellofemoral cartilage lesions, this technique has yet to be described in the literature for large traumatic osteochondral loss of a lateral femoral trochlea [4,5]. We report this case of a 22-year-old female with patellofemoral issues secondary to significant osteochondral loss of the lateral femoral trochlea who underwent an OCA procedure of the lateral femoral trochlea.

## Case presentation

**Figure 1:**
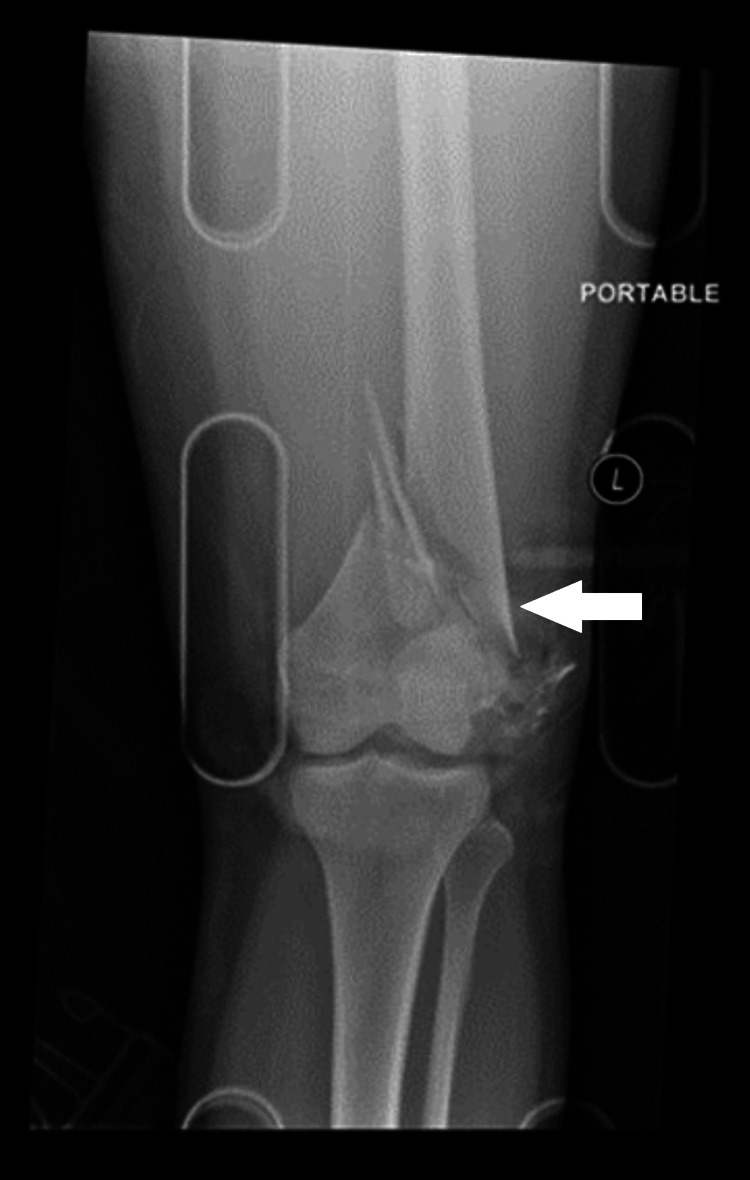
Initial injury AP X-ray image of the left knee revealing a comminuted fracture of the distal femur involving the distal diaphysis, which is shortened and extends laterally through the femoral condyle, as highlighted by the arrow. AP: Anteroposterior.

**Figure 2:**
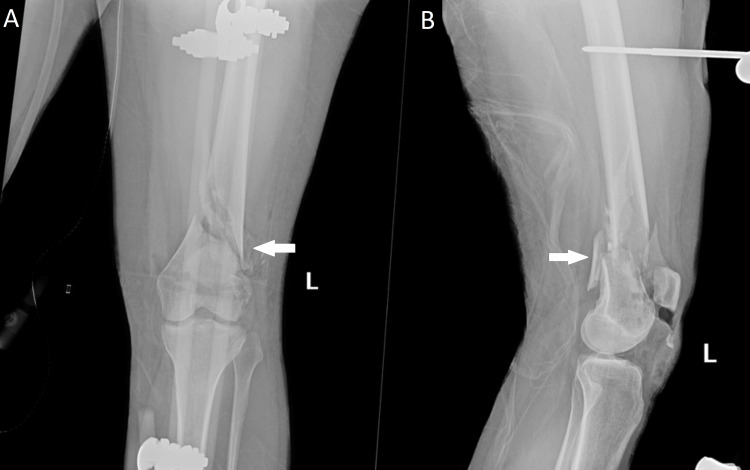
Postoperative external fixation procedure: (A) AP and (B) lateral images of the left knee revealing a knee-spanning external fixator in place and a comminuted distal femur fracture that is restored to length, as highlighted by the arrows. AP: Anteroposterior.

**Figure 3:**
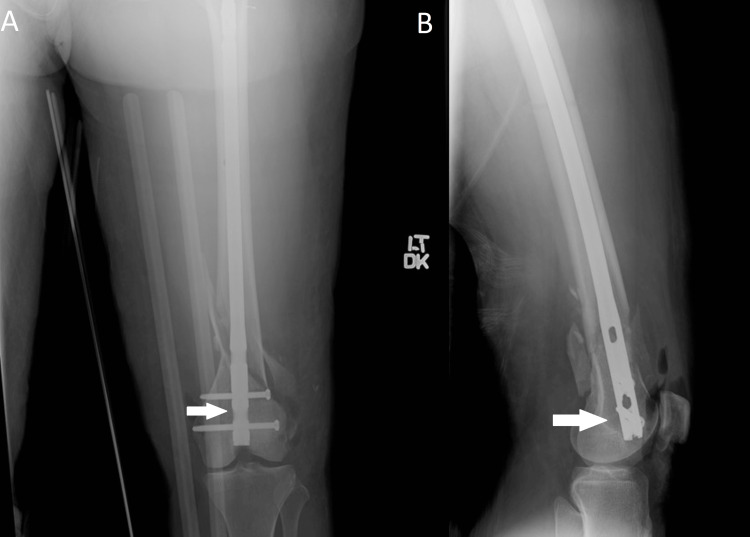
Postoperative retrograde intramedullary nailing: (A) AP and (B) lateral images of the left femur revealing a retrograde intramedullary nail in good position, as highlighted by the arrows. AP: Anteroposterior.

**Figure 4:**
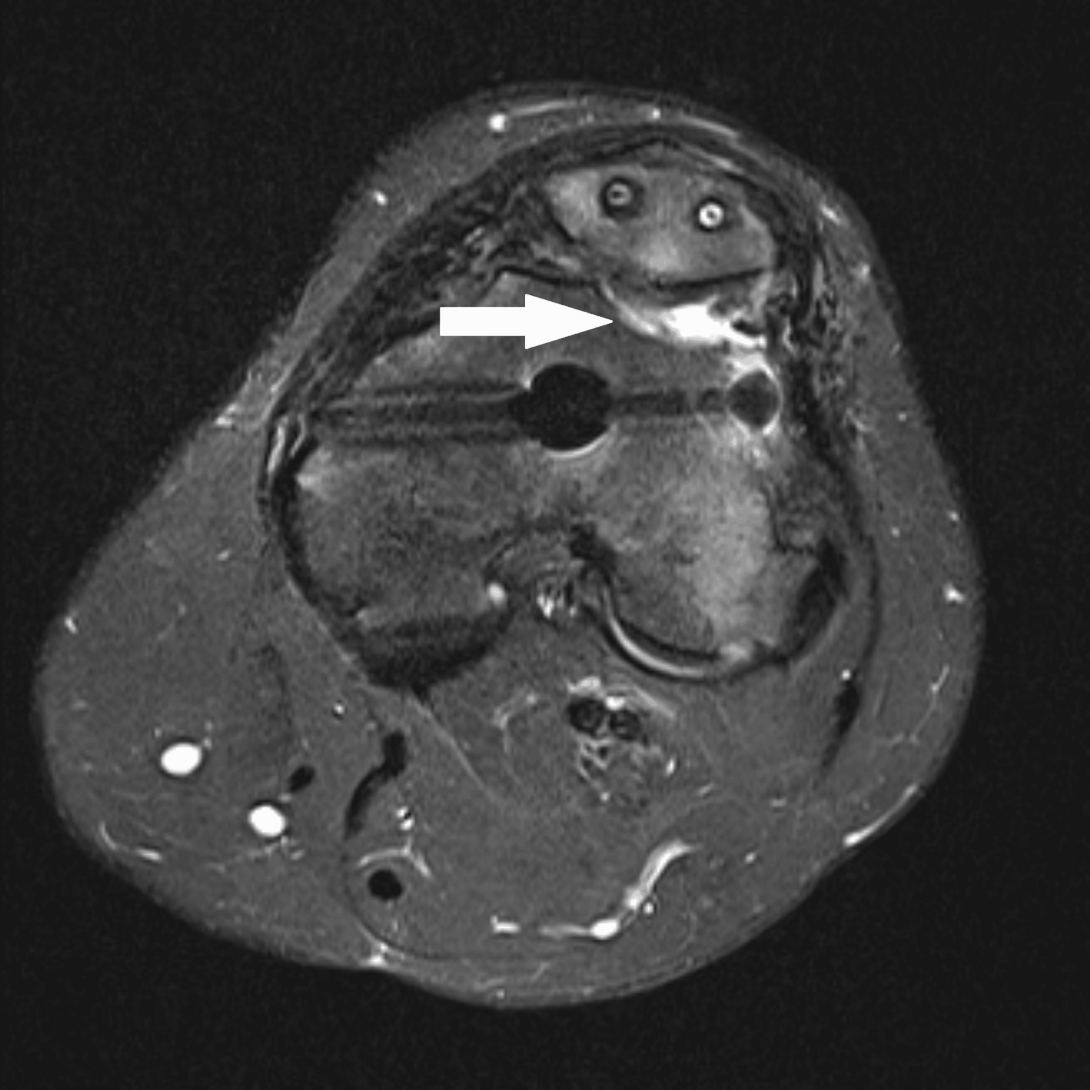
Pre-operative axial cut of short tau inversion recovery (STIR) MRI of the left distal femur demonstrating mild lateral patellar subluxation with thickening, intermediate signal of the patellar retinaculum, and lateral patellofemoral chondromalacia, as highlighted by the arrow.

A 23-year-old previously healthy female patient was involved in a motor vehicle collision (MVC) where she sustained multiple injuries, including a left open distal femur fracture with significant comminution of the lateral trochlea (Figure 1). She underwent irrigation and debridement and placement of a knee-spanning external fixator (ex-fix) at the index procedure (Figure 2). Eight days later, she underwent removal of the ex-fix and placement of a retrograde femoral intramedullary nail with patellar tendon repair (Figure 3). The patient continued to complain of knee pain at her six-month follow-up. Eight months post-operatively, she was seen by an outside orthopedic surgeon for ongoing knee pain. Magnetic resonance imaging was obtained, which demonstrated mild lateral patellar subluxation with thickening, intermediate signal of the patellar retinaculum, and lateral patellofemoral chondromalacia (Figure 4).

She presented for a second opinion. She complained of recurrent painful lateral subluxation of her left patella with knee flexion, along with feeling unstable while walking. On examination, there was a good ROM of the left knee, significant patellar crepitus, lateral patellar tracking, and a large clunk felt at 30 degrees of flexion. We discussed the possibility of surgical fixation focused around a cadaveric allograft replacement of at least the lateral aspect of the patellofemoral joint if not the entire patellofemoral joint, with the patient in agreement.

**Figure 5:**
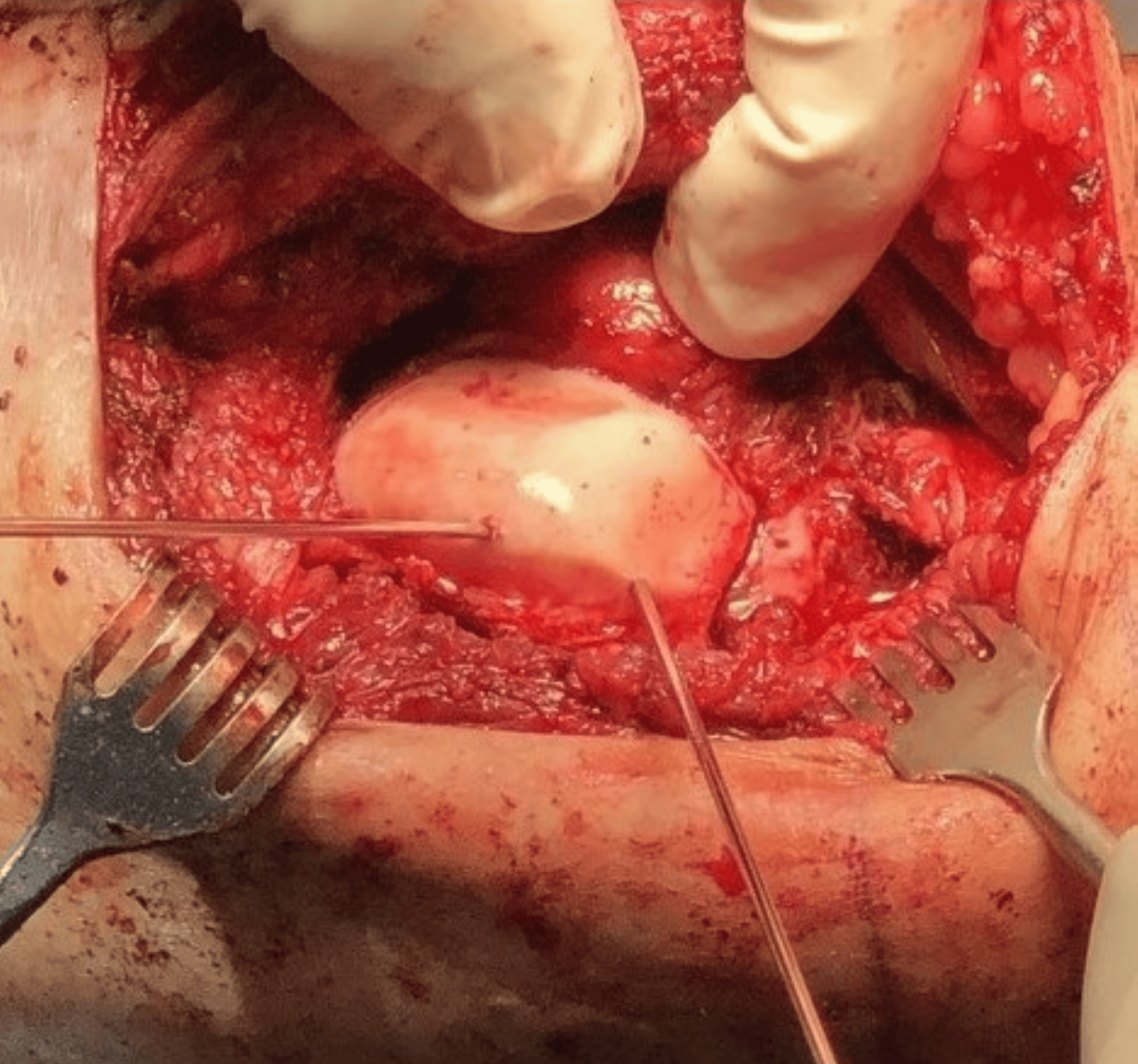
Intraoperative photo of the left distal femur revealing a lateral trochlea osteochondral allograft held in place with provisional K-wire fixation.

**Figure 6:**
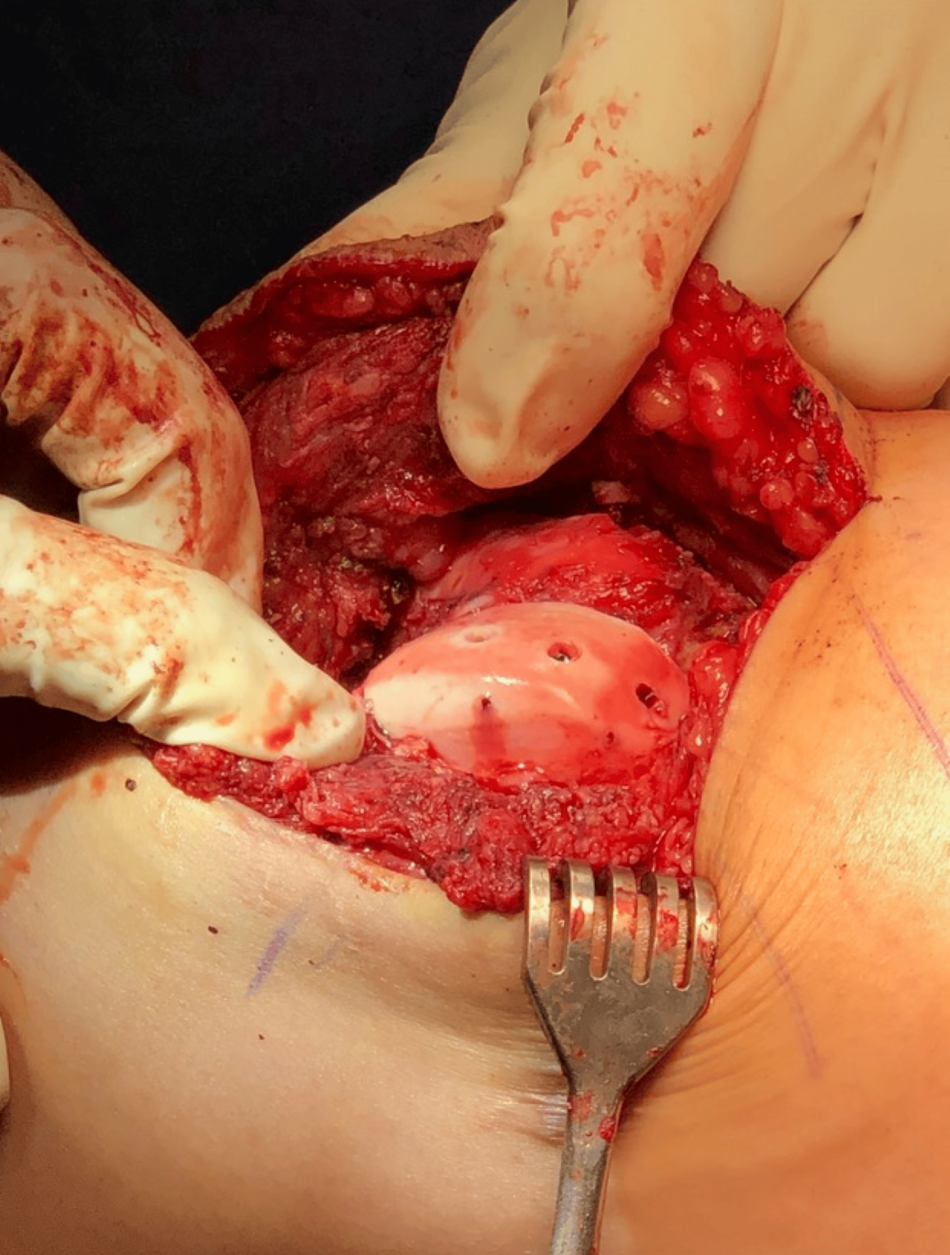
Intraoperative photo of the left distal femur revealing three 3.5 mm headless compression screws in place in the lateral femoral trochlea osteochondral allograft.

We proceeded with surgical intervention one year after the initial injury. The patient received a regional pain block, was placed under general anesthesia, and the left lower extremity was prepped and draped with a sterile tourniquet placed. A skin incision was made incorporating the previous infrapatellar surgical scar, extending it proximally and laterally. A lateral parapatellar approach was used, and the patella was inverted medially. An incision was made in the suprapatellar pouch along the lateral aspect of the trochlea, and a significant trochlear defect was appreciated. The distal locking screw of the intramedullary fixation was removed as it was going to be in the way of the placement of the donor graft. A recipient bed was made for the allograft using a small sagittal saw and cutting from the mid trochlear groove laterally. The allograft was then fashioned to fit that defect nearly perfectly, measuring 4.3cm x 2.8cm x 1.6cm. The allograft was held in place with provisional K-wire fixation, and radiographs showed a proper fit (Figure 5). Three 3.5 mm headless compression screws were placed over appropriate K-wire placement, with appropriate position and compression noted radiographically (Figure 6). A dovetail notch was made in the metaphyseal area, just proximal to the lateral trochlea to keep the graft from sliding proximally under stress. The knee was put through range of motion (ROM) and the patella was noted to track appropriately. The lateral parapatellar arthrotomy was closed using absorbable suture followed by a platelet-rich plasma injection into the joint near the lateral trochlea. The subcutaneous and cutaneous layers were then closed with skin dressings placed on top.

**Figure 7:**
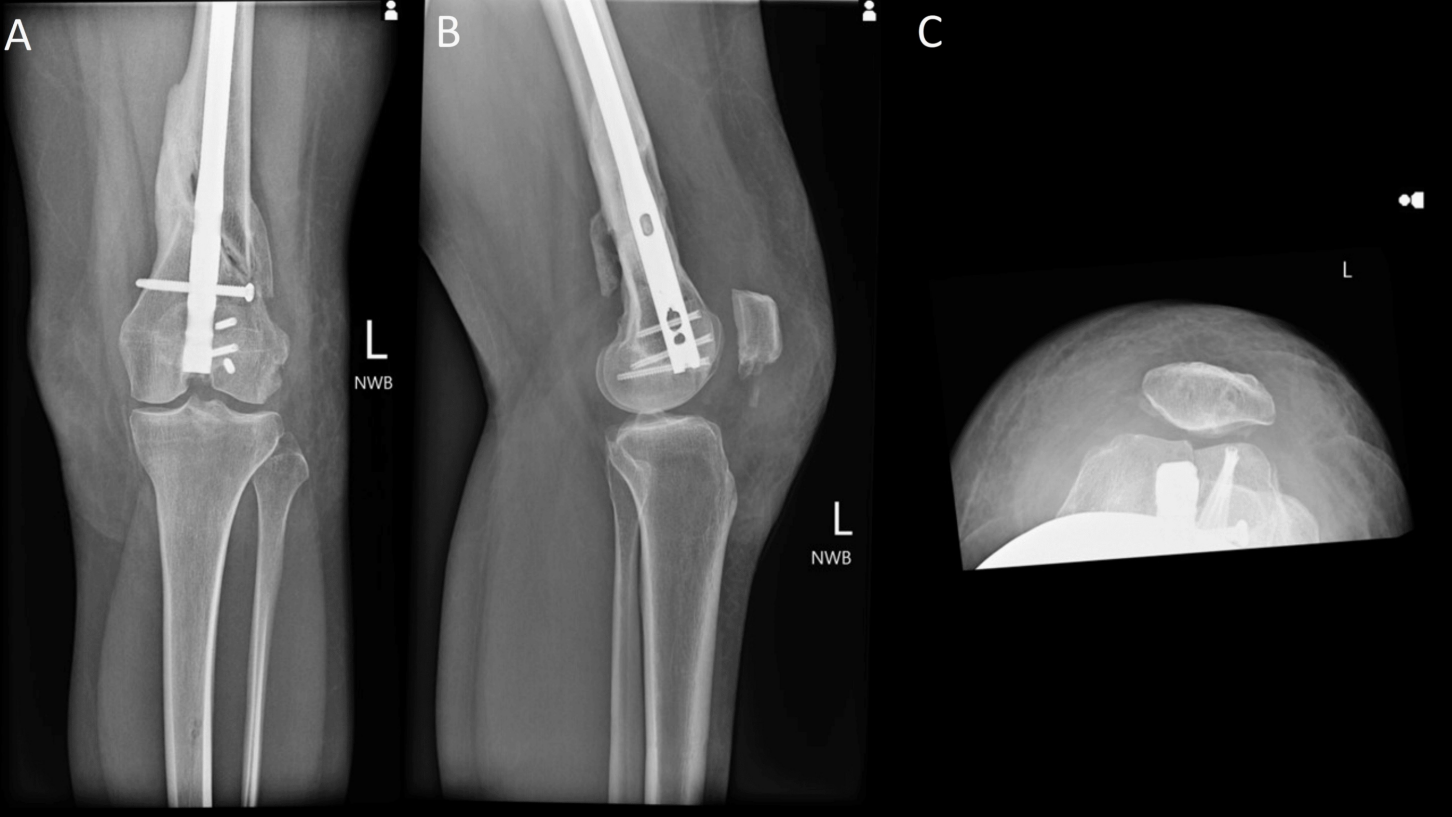
One week postoperative osteochondral allograft transplant: (A) AP, (B) lateral, and (C) sunrise views of the left knee revealing the allograft reconstruction of the left lateral trochlea in good position without evidence of compromise or hardware failure. AP: Anteroposterior.

One week later, she complained of swelling and pain (4-5/10) and reported standing on her left leg for a couple of hours each day but had not moved her knee much. She confirmed she was elevating it often to help with swelling. On examination, she had a well-healed incision, moderate swelling of the left knee, active ROM from 5 to 40 degrees, and was neurovascularly intact. Radiographs showed the allograft reconstruction of the left lateral trochlea was in good position without evidence of compromise or hardware failure (Figure 7). The plan at that time was to continue with ice and elevation and to restrict movement to 30 degrees of flexion out of brace. At two weeks post-op, she was doing well. On examination, the incision was well healed, passive/active ROM was 0 to 90 degrees flexion, and she was neurovascularly intact. The plan was to weight bear as tolerated, restricted only to deep squats and knee bending moments, and to begin ROM home exercises.

**Figure 8:**
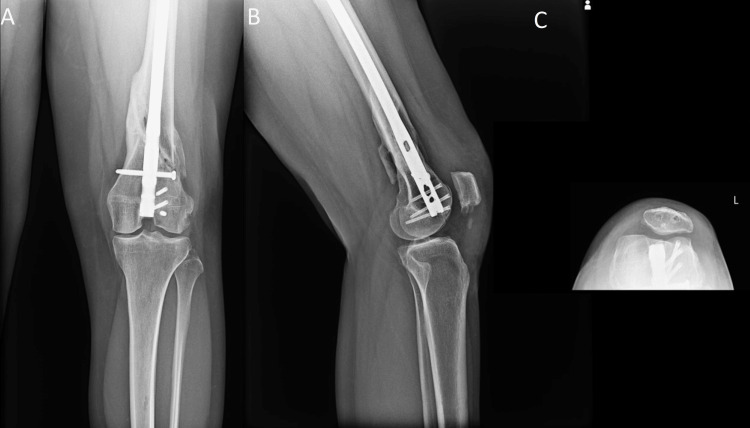
Two months postoperative osteochondral allograft transplant: (A) AP, (B) lateral, and (C) sunrise views of the left knee revealing the allograft reconstruction of the lateral trochlea healing in a near-anatomic position with hardware in good position. AP: Anteroposterior.

At the 2-month follow-up, she reported an improved gait and completing stairs. On examination, a mild extensor lag of the left knee was appreciated with ROM, a well-healed incision, normal gait, no instability of the left patellofemoral joint, and she was neurovascularly intact. Radiographs showed the allograft reconstruction of the lateral trochlea healing in a near-anatomic position with hardware in good position (Figure 8). We began activity as tolerated and continued home exercises.

**Figure 9:**
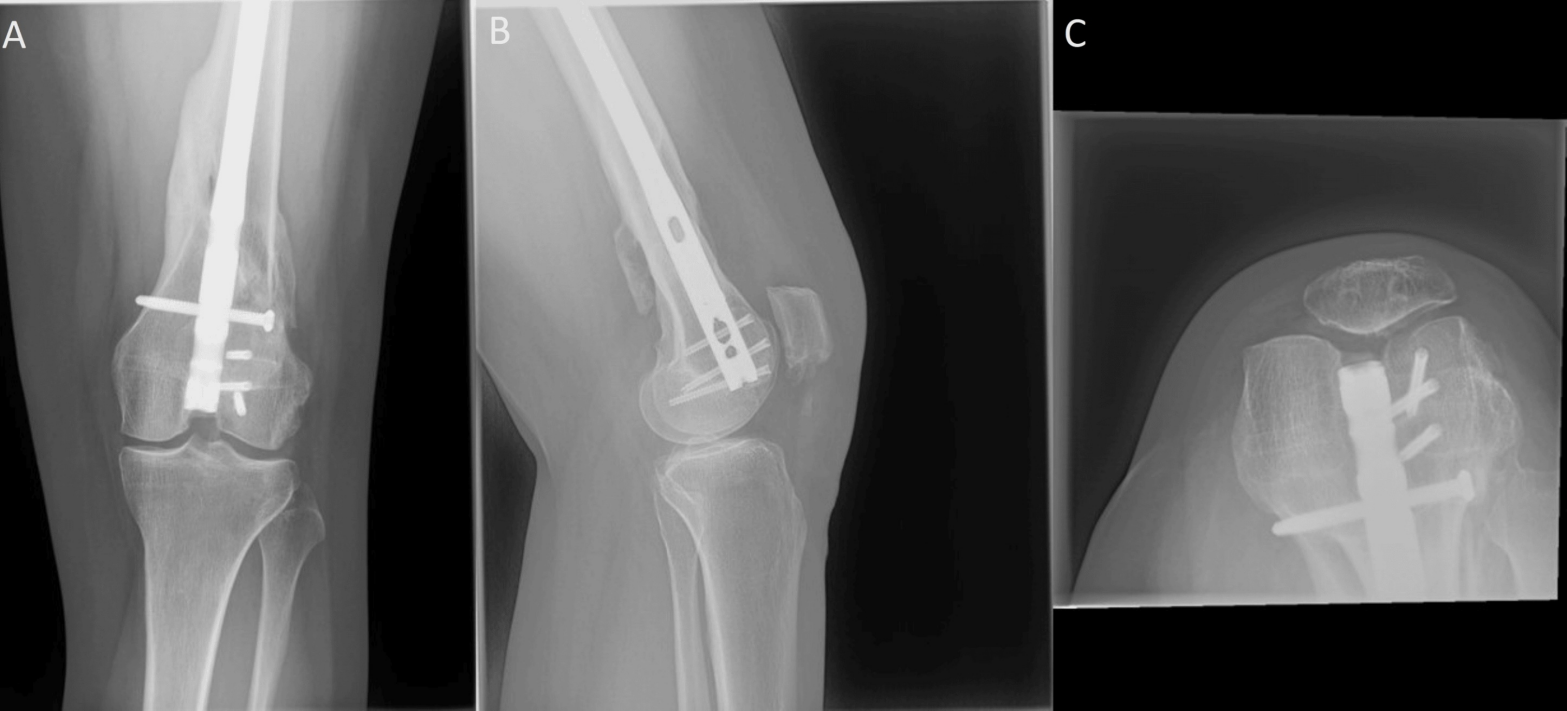
Six months postoperative osteochondral allograft transplant: (A) AP, (B) lateral, and (C) sunrise views of the left knee revealing no interval change since the previous radiographs. AP: Anteroposterior.

At six months, she had done more walking than usual, had mild left knee pain, and had some difficulty getting in and out of a car and with stairs, but could ambulate and do everything she needed to. On examination, the left knee had nearly full ROM, good patellar tracking, no patellar grinding, and was neurovascularly intact. Radiographs of the left knee showed no change since the last imaging (Figure 9). At 4 years post-op, she had full extension, a slight flexion deficit compared to the right that is only noticed when squatting, a Visual Analog Scale (VAS) score of 1, and a Knee Injury and Osteoarthritis Outcome Score (KOOS-12) of 77. The patient was happy she underwent the procedure and felt she had improved considerably. The patient was informed that data concerning the case would be submitted for publication, and she provided consent.

## Discussion

Potential surgical interventions for this patient’s patellofemoral instability included trochleoplasty vs OCA vs osteochondral autograft transplantation procedure. We believed trochleoplasty would not benefit the patient due to the lasting defect of the lateral femoral trochlea after the previous trauma and the patient not meeting the indications, including Type B or D dysplasia (DeJour). When comparing allografts versus autografts, osteochondral allografts resulted in a nearly equivalent graft survival rate and had an average of 16% higher patient-reported outcomes than autografts [6]. Other benefits of allograft use include the immediate filling of the chondral defect with mature articular cartilage, the avoidance of donor site morbidity associated with autograft harvest, and a low likelihood of rejection by host tissue due to its immunologically inert nature [7-9]. The literature surrounding OCA procedures showed promise as a potential long-term treatment for this patient.

Patients who underwent femoral trochlea OCA due to a patellofemoral cartilage lesion reported improved pain and function at 7 years [5]. An OCA of the distal femur was found to be a long-term solution for large articular defects in young and active patients, with results showing a mean modified Hospital for Special Surgery (HSS) score of 86 at 15 years [10]. There is a gap in the literature regarding an OCA procedure of the lateral femoral trochlea due to traumatic injury rather than congenital deformity, dysplasia, or lesions. Although the etiology of trochlear dysfunction is different, the patient was still experiencing patellofemoral instability due to abnormal anatomy. We believed that an OCA procedure could provide a similar benefit to our patient.

After confirmation of the OCA procedure with the patient, obtaining a donor graft took some time. Others should educate their patients about a timeline of several weeks to months for donor graft availability. A platelet-rich plasma (PRP) injection into the joint was performed to promote healing and integration of the allograft. There is evidence that the integration of adjacent cartilage surfaces is improved at three weeks with a PRP injection compared to no injection [11]. We felt that three evenly spaced headless compression screws held the graft in place effectively. The patient tolerated the procedure well and was able to return to all daily activities without issue at the six-month mark. Future research should focus on the long-term follow-up of patients undergoing an OCA procedure of the lateral femoral trochlea due to traumatic etiology.

## Conclusions

Osteochondral allograft transplantation should be evaluated as a possible treatment option for patients presenting with a large traumatic lateral trochlear defect, especially in those who experience painful patellofemoral instability. This case demonstrates that good subjective and objective outcomes can be expected at mid-term follow-up.
